# Supplementary material for: In vivo CRISPR screening identifies NF1/RASA1/TP53 co-mutations and downstream MEK signaling as a common key mechanism of sinonasal tumorigenesis
Source: bioRxiv. 2025 Jun 24:2025.05.19.654661. Originally published 2025 May 23. Preprint. [Version 2] doi: 10.1101/2025.05.19.654661 (PMC12139907; doi:10.1101/2025.05.19.654661)
Supplement: 1 [file NIHPP2025.05.19.654661V2-supplement-1.pdf]

bioRxiv preprint doi: https://doi.org/10.1101/2025.05.19.654661; this version posted June 24, 2025. The copyright holder for this preprint (which was not certified by peer review) is the author/funder. This article is a US Government work. It is not subject to copyright under 17 USC 105 and is also made available for use under a CC0 license.

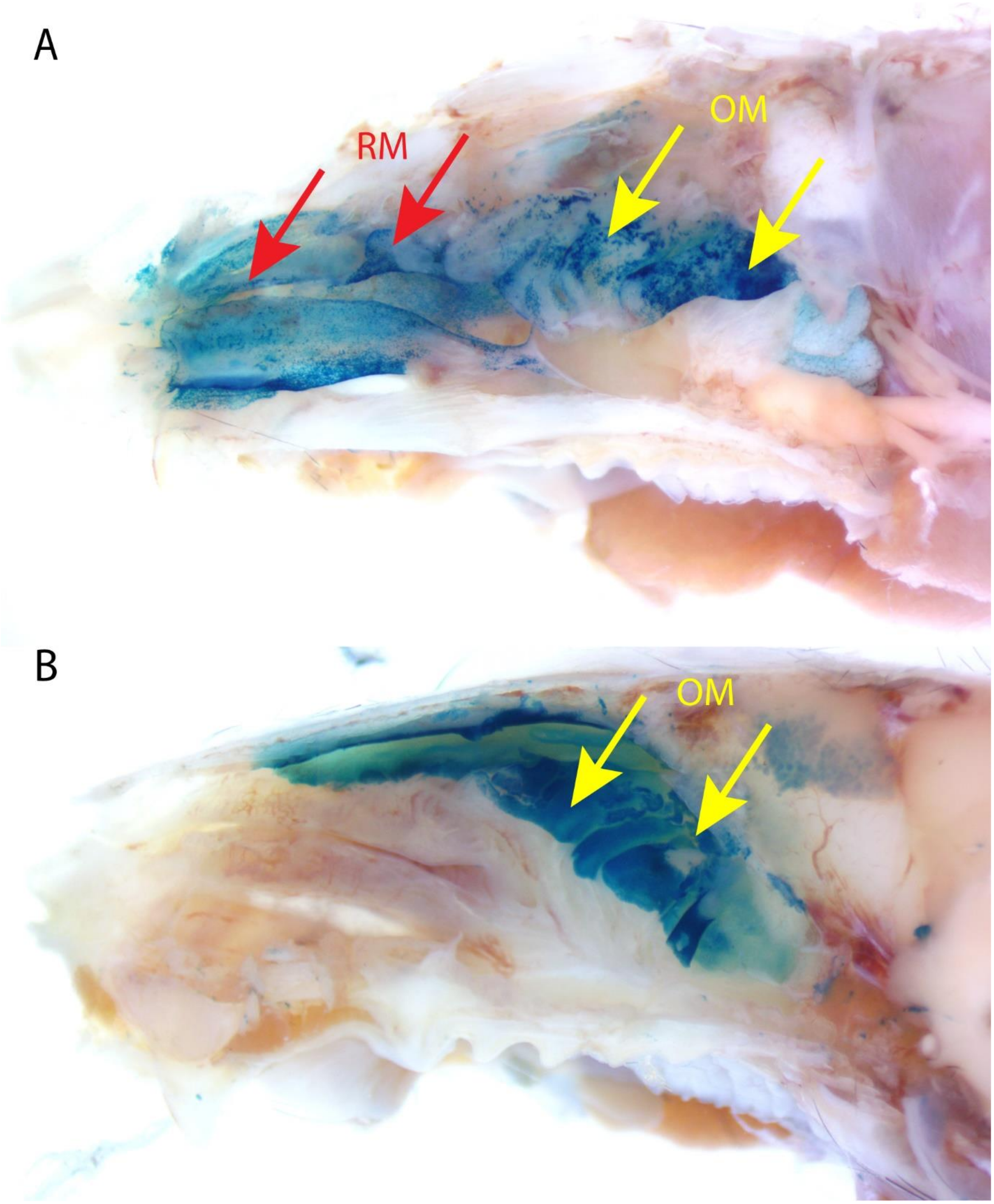

Supplementary Figure 1. AAV5-Cre broadly transduces sinonasal cells across the olfactory and respiratory epithelium. (A) Sinonasal instillation of AAV5-Cre was performed in R26R mice. X-gal staining (blue) reveals representative transduction across the olfactory mucosa (OM, yellow arrows) and the respiratory mucosa (RM, red arrows). **(B)** For reference, OMP-Cre mice were crossed with R26R mice and X-gal staining demonstrates the location of the olfactory mucosa (yellow arrows).

bioRxiv preprint doi: https://doi.org/10.1101/2025.05.19.654661; this version posted June 24, 2025. The copyright holder for this preprint (which was not certified by peer review) is the author/funder. This article is a US Government work. It is not subject to copyright under 17 USC 105 and is also made available for use under a CC0 license.

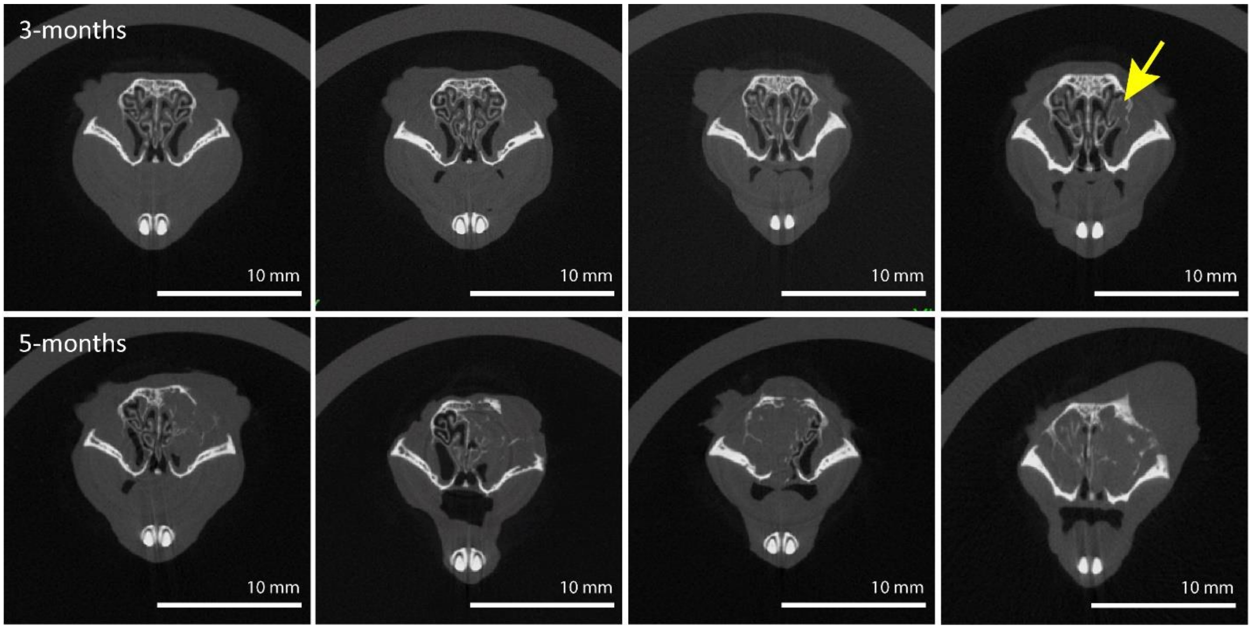

Supplementary Figure 2. Sinonasal tumor formation after AAV5-*TP53*-sgRNA library instillation in H11^{Cas9} mice on microCT. Thirty-three H11^{Cas9} were exposed to the AAV5-*TP53*-sgRNA library. Initial signs of tumor formation were noted as early as 3 months post-instillation on microCT **(top row)** with large tumor formation noted around 5 months **(bottom row)**. Twenty-eight of these thirty-three H11^{Cas9} mice developed a sinonasal tumor.

bioRxiv preprint doi: https://doi.org/10.1101/2025.05.19.654661; this version posted June 24, 2025. The copyright holder for this preprint (which was not certified by peer review) is the author/funder. This article is a US Government work. It is not subject to copyright under 17 USC 105 and is also made available for use under a CC0 license.

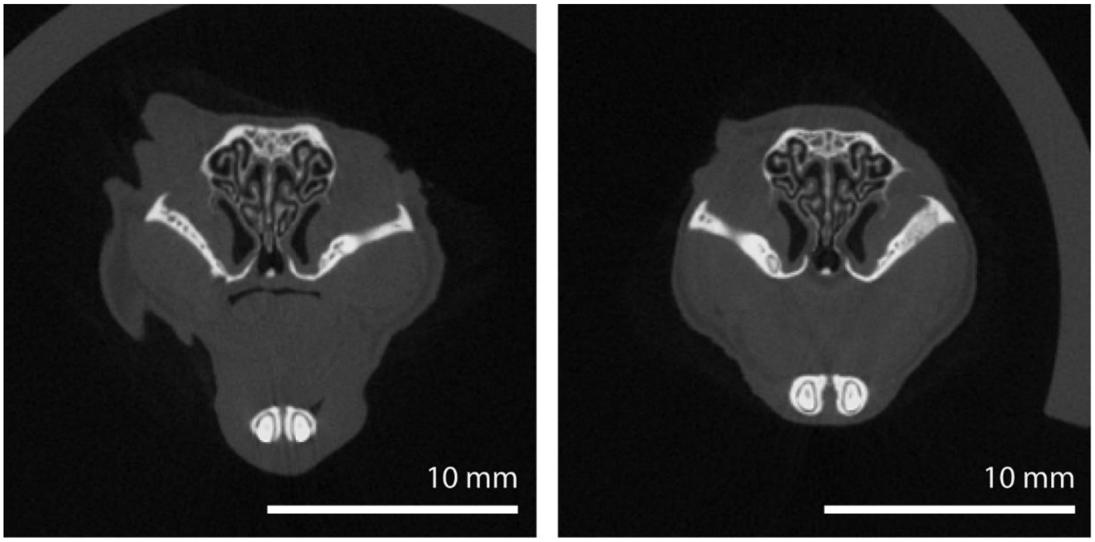

Supplementary Figure 3. No sinonasal tumor formation observed after AAV5-*TP53*-null instillation in H11^{Cas9} mice on microCT. Twenty-seven H11^{Cas9} mice were exposed to AAV5-*TP53*-null control virus. No tumor formation was observed on microCT within 16 months after administration in any of these control mice. Representative coronal images from two mice are shown here.

bioRxiv preprint doi: https://doi.org/10.1101/2025.05.19.654661; this version posted June 24, 2025. The copyright holder for this preprint (which was not certified by peer review) is the author/funder. This article is a US Government work. It is not subject to copyright under 17 USC 105 and is also made available for use under a CC0 license.

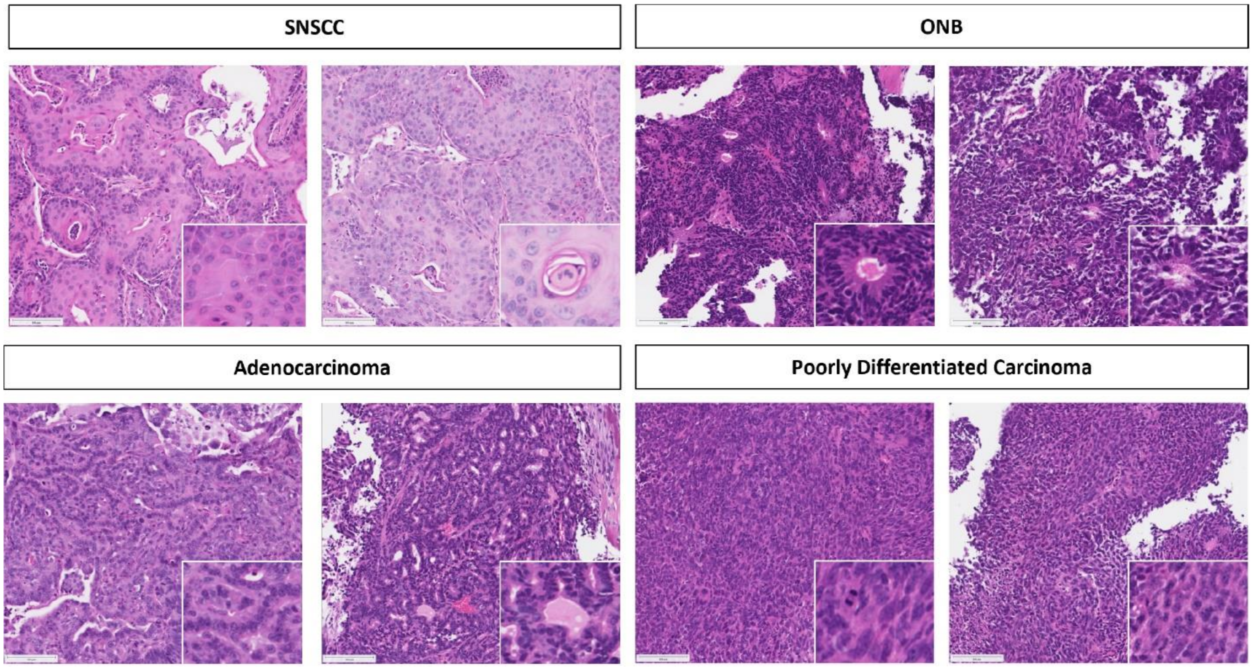

Supplementary Figure 4. Histologic demonstration of murine sinonasal tumor types. Hematoxylin & Eosin staining demonstrated histologic findings consistent with SNSCC, ONB, adenocarcinoma, and poorly differentiated carcinoma. Representative images from two tumors from each tumor type are shown along with high magnification inset of key pathologic features. Scalebar equals 50µm. SNSCC – sinonasal squamous cell carcinoma, ONB – olfactory neuroblastoma.

bioRxiv preprint doi: https://doi.org/10.1101/2025.05.19.654661; this version posted June 24, 2025. The copyright holder for this preprint (which was not certified by peer review) is the author/funder. This article is a US Government work. It is not subject to copyright under 17 USC 105 and is also made available for use under a CC0 license.

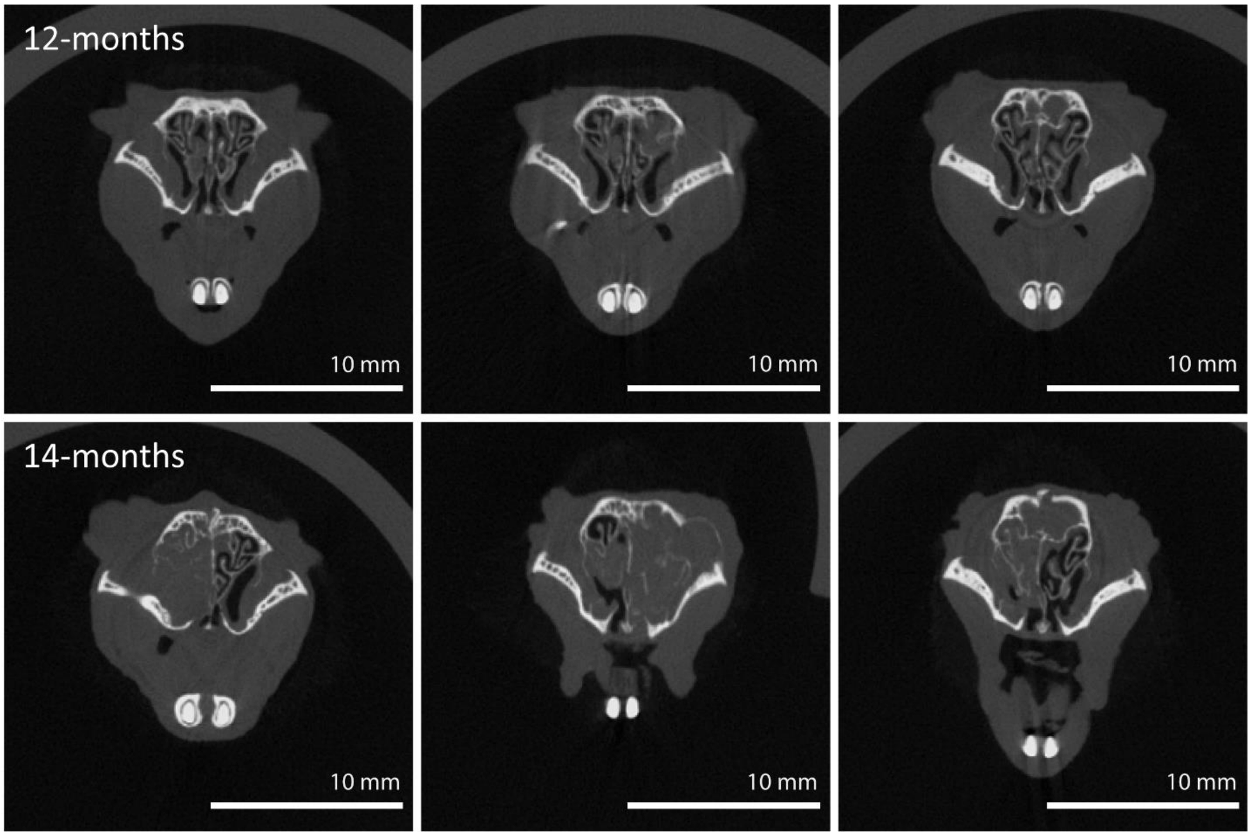

Supplementary Figure 5. Sinonasal tumor formation after AAV5-Cre installation in *NF1/TP53*$^{flox/flox}$ mice on microCT. Twenty *NF1/TP53*$^{flox/flox}$ mice were exposed to AAV5-Cre. Initial signs of tumor formation were noted around 12 months post-instillation on microCT **(top row)** with slow growth noted around 14 months **(bottom row)**. Thirteen of these twenty *NF1/TP53*$^{flox/flox}$ mice developed a sinonasal tumor.

bioRxiv preprint doi: https://doi.org/10.1101/2025.05.19.654661; this version posted June 24, 2025. The copyright holder for this preprint (which was not certified by peer review) is the author/funder. This article is a US Government work. It is not subject to copyright under 17 USC 105 and is also made available for use under a CC0 license.

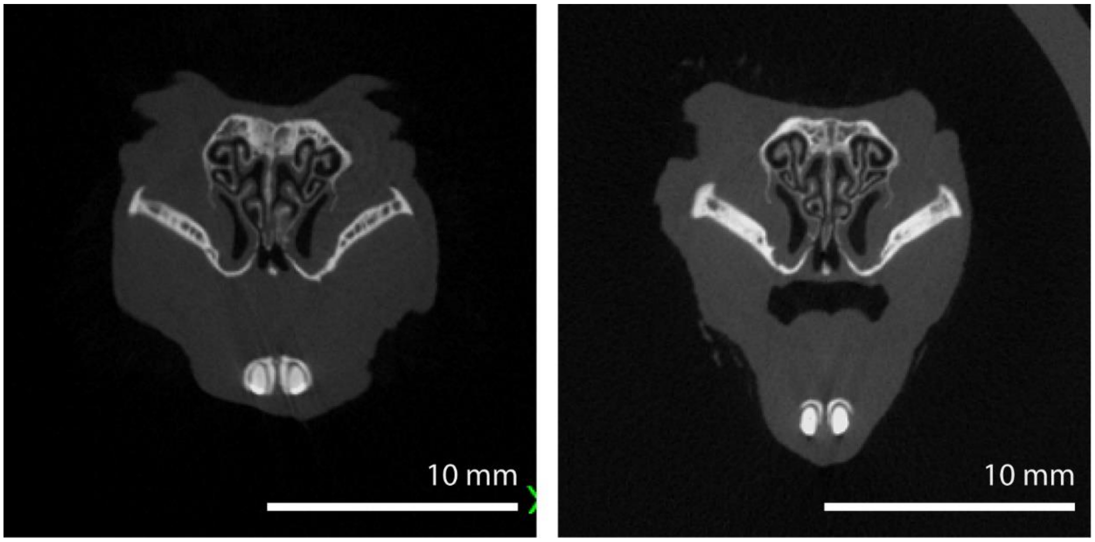

Supplementary Figure 6. No sinonasal tumor formation observed after AAV5-null instillation in *NF1/TP53*$^{flox/flox}$ mice on microCT. Fifteen *NF1/TP53*$^{flox/flox}$ mice were exposed to AAV5-null control virus. No tumor formation was observed on microCT within 16 months after administration in any of these control mice. Representative coronal images from two mice are shown here.

bioRxiv preprint doi: https://doi.org/10.1101/2025.05.19.654661; this version posted June 24, 2025. The copyright holder for this preprint (which was not certified by peer review) is the author/funder. This article is a US Government work. It is not subject to copyright under 17 USC 105 and is also made available for use under a CC0 license.

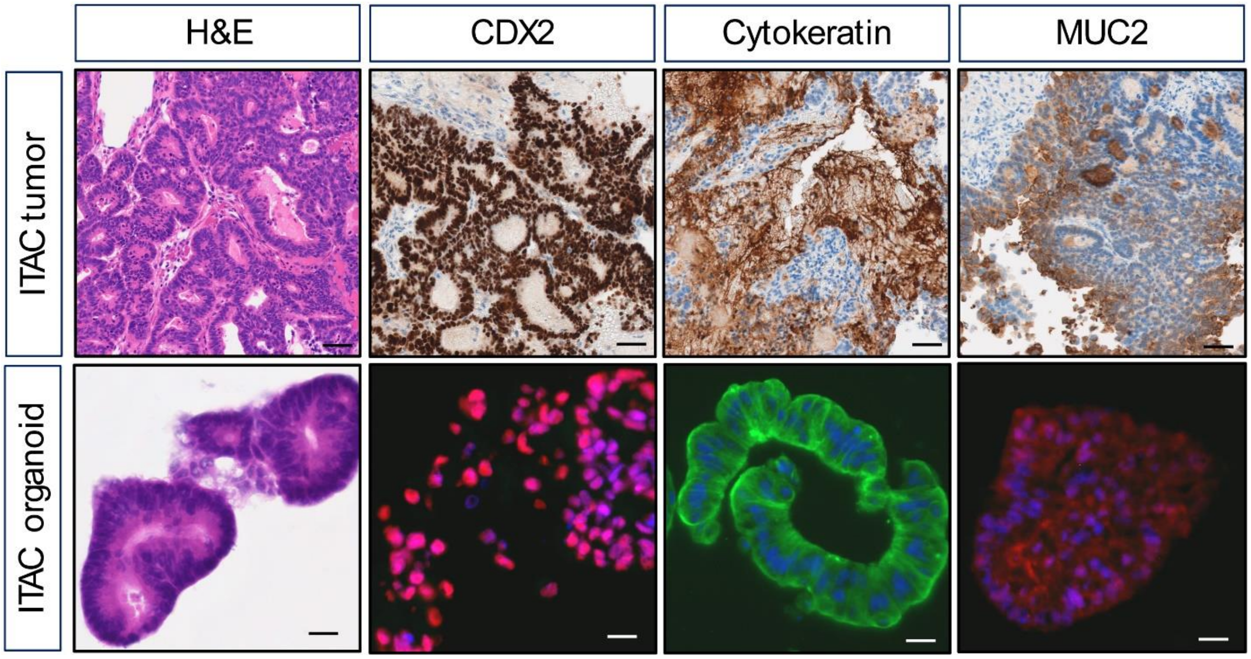

Supplementary Figure 7. Histological characterization of human sinonasal intestinal type adenocarcinoma tumor tissue and organoids. (Top row) Human sinonasal intestinal type adenocarcinoma (ITAC) tumor tissue from which organoids were derived demonstrate histologic patterns and expression of markers of sinonasal ITAC including CDX2, cytokeratin, and MUC2. **(Bottom row)** Sinonasal ITAC organoids demonstrate histologic characteristics and expression of key markers including CDX2, cytokeratin, and MUC2. Scale bars for H&E and immunohistochemistry are 20 μM while scale bars in immunofluorescence are 200 μM.

bioRxiv preprint doi: https://doi.org/10.1101/2025.05.19.654661; this version posted June 24, 2025. The copyright holder for this preprint (which was not certified by peer review) is the author/funder. This article is a US Government work. It is not subject to copyright under 17 USC 105 and is also made available for use under a CC0 license.

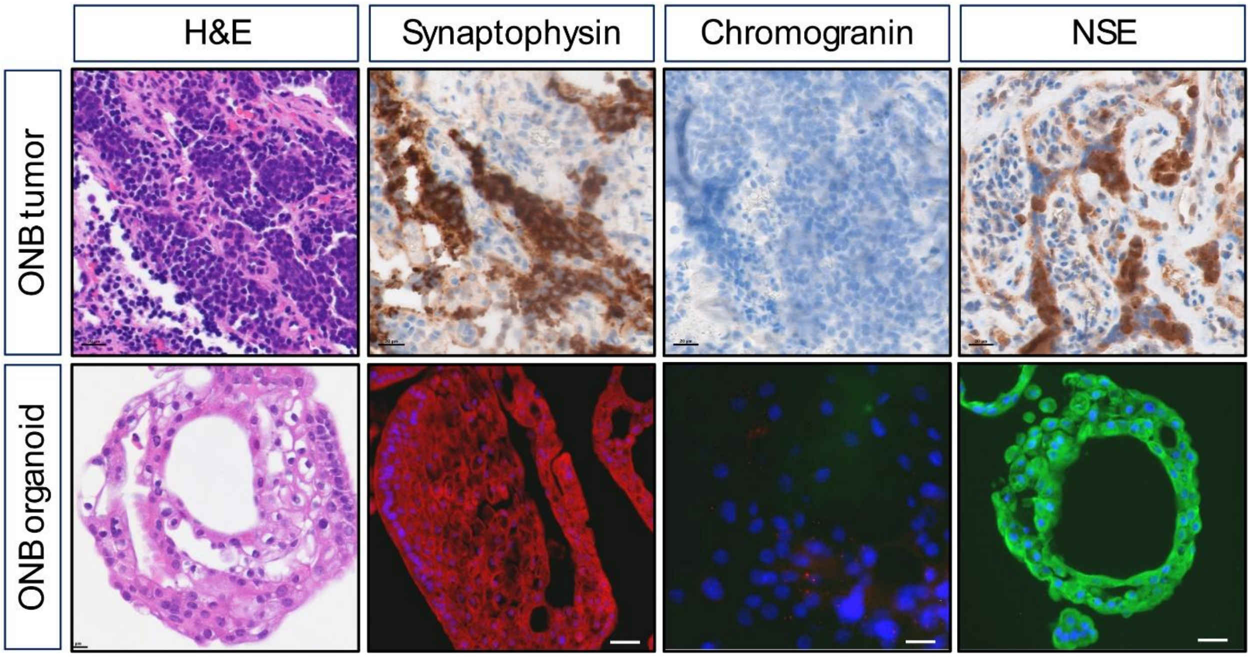

Supplementary Figure 8. Histological characterization of human ONB tumor tissue and organoids. (Top row) Human olfactory neuroblastoma (ONB) tumor tissue from which organoids were derived demonstrate histologic patterns and expression of markers of ONB including synaptophysin and NSE. This particular tumor had low chromogranin expression. **(Bottom row)** ONB organoids demonstrate histologic characteristics and expression of key markers including synaptophysin and NSE with low chromogranin expression as seen in the primary tumor sample. Scale bars for H&E and immunohistochemistry are 20 μM while scale bars in immunofluorescence are 200 μM.

bioRxiv preprint doi: https://doi.org/10.1101/2025.05.19.654661; this version posted June 24, 2025. The copyright holder for this preprint (which was not certified by peer review) is the author/funder. This article is a US Government work. It is not subject to copyright under 17 USC 105 and is also made available for use under a CC0 license.

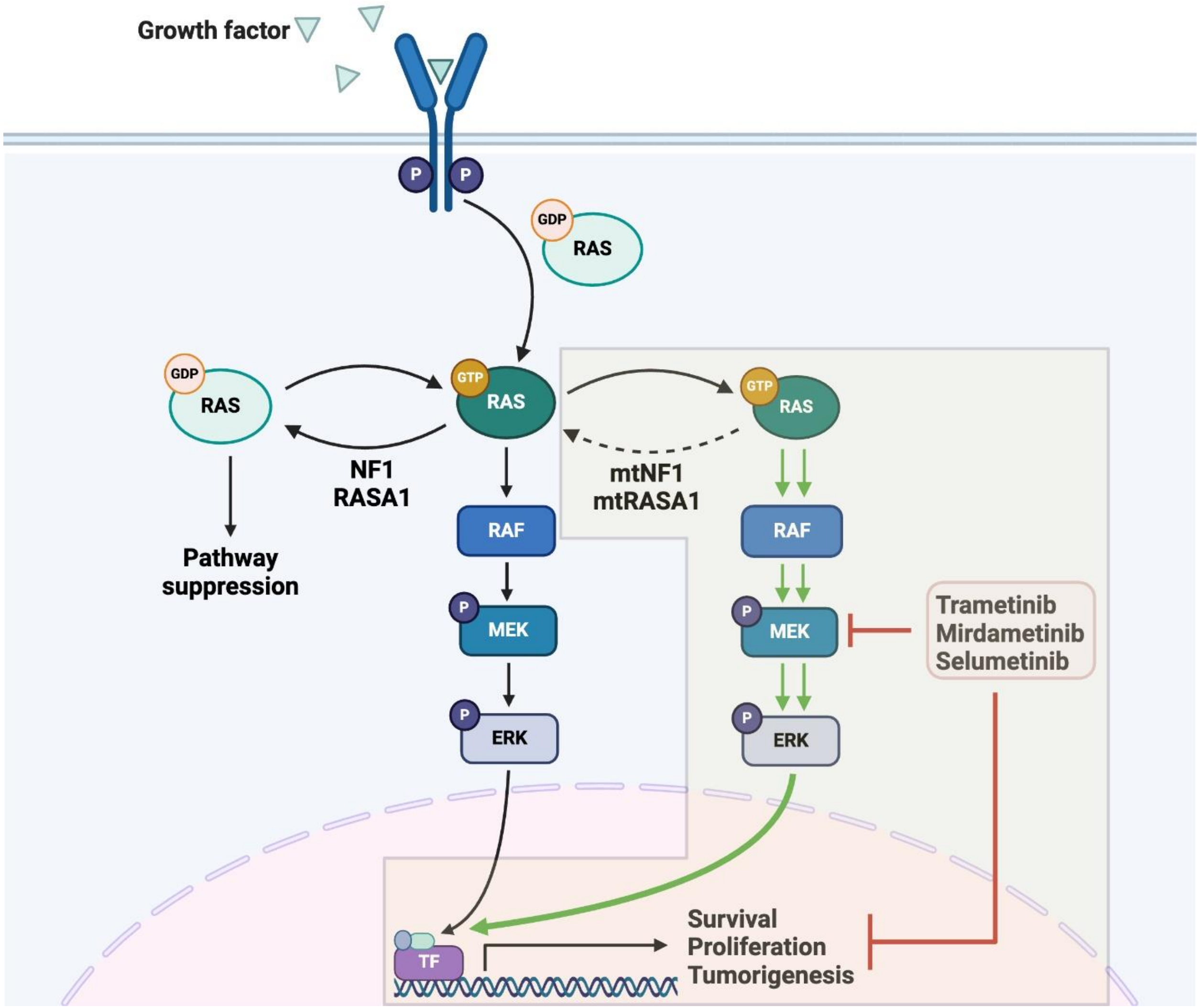

Supplementary Figure 9. Loss of NF1 and RASA1-mediated Ras-GAP activity leads to Ras activation and downstream MEK signaling. Therefore, targeting MEK signaling with small molecule inhibitors such as Trametinib, Mirdametinib, and Selumetinib may be a potential common target in major sinonasal tumor subtypes.

bioRxiv preprint doi: https://doi.org/10.1101/2025.05.19.654661; this version posted June 24, 2025. The copyright holder for this preprint (which was not certified by peer review) is the author/funder. This article is a US Government work. It is not subject to copyright under 17 USC 105 and is also made available for use under a CC0 license.

Supplementary Table 1. List of sgRNA targets in the AAV5-*TP53*-sgRNA library.

Supplementary Table 2. Summary of genes with a lower frequency of sgRNA perturbation.
